# Genetic Analysis of Mutagenesis That Induces the Photoperiod Insensitivity of Wild Cotton *Gossypium hirsutum* Subsp. *purpurascens*

**DOI:** 10.3390/plants11223012

**Published:** 2022-11-08

**Authors:** Fakhriddin N. Kushanov, Doniyor J. Komilov, Ozod S. Turaev, Dilrabo K. Ernazarova, Roza S. Amanboyeva, Bunyod M. Gapparov, John Z. Yu

**Affiliations:** 1 Institute of Genetics and Plant Experimental Biology, Academy of Sciences of the Republic of Uzbekistan, Qibray QFY, Yuqori-Yuz, Qibray District, Tashkent 111226, Uzbekistan; 2 Department of Biology, National University of Uzbekistan, University Street-4, Olmazor District, Tashkent 100174, Uzbekistan; 3 Department of Biotechnology, Namangan State University, Uychi Street-316, Namangan 160100, Uzbekistan; 4 Center of Genomics and Bioinformatics, Academy of Sciences of the Republic of Uzbekistan, University Street-2, Qibray District, Tashkent 111215, Uzbekistan; 5 Faculty of Natural Sciences, Gulistan State University, 4th Microregion, Gulistan 120100, Uzbekistan; 6 United States Department of Agriculture (USDA)-Agricultural Research Service (ARS), Southern Plains Agricultural Research Center, 2881 F&B Road, College Station, TX 77845, USA

**Keywords:** cotton (*Gossypium*), mutagenesis, photoperiod sensitivity, quantitative trait locus (QTL), simple sequence repeat (SSR)

## Abstract

Cotton genus *Gossypium* L., especially its wild species, is rich in genetic diversity. However, this valuable genetic resource is barely used in cotton breeding programs. In part, due to photoperiod sensitivities, the genetic diversity of *Gossypium* remains largely untapped. Herein, we present a genetic analysis of morphological, cytological, and genomic changes from radiation-mediated mutagenesis that induced plant photoperiod insensitivity in the wild cotton of *Gossypium hirsutum*. Several morphological and agronomical traits were found to be highly inheritable using the progeny between the wild-type *G. hirsutum* subsp. *purpurascens* (El-Salvador) and its mutant line (Kupaysin). An analysis of pollen mother cells (PMCs) revealed quadrivalents that had an open ring shape and an adjoining type of divergence of chromosomes from translocation complexes. Using 336 SSR markers and 157 F_2_ progenies that were grown with parental genotypes and F_1_ hybrids in long day and short night conditions, five quantitative trait loci (QTLs) associated with cotton flowering were located on chromosomes At-05, At-11, and Dt-07. Nineteen candidate genes related to the flowering traits were suggested through molecular and in silico analysis. The DNA markers associated with the candidate genes, upon future functional analysis, would provide useful tools in marker-assisted selection (MAS) in cotton breeding programs for early flowering and maturity.

## 1. Introduction

The cotton genus (*Gossypium* L.) belongs to the taxonomic tribe *Gossypieae* (order *Malvales*; family *Malvaceae*) with nine genera in total, eight of which are classically recognized [1,2]. The genus *Gossypium* has a polytopic-monophyletic origin [3]. Many species of the genus arose at the end of the Tertiary period and were distributed in three very different subgenera. They represent three phylogenetic horns: the New World (*Karpas*), the Old World (*Eugossypium*), and Australia (*Sturtia*). The Karpas formed at the end of the Cretaceous when the New World landmass broke apart [3].

Currently, there are at least 50 *Gossypium* species belonging to nine genome groups (A-G, K, and AD genomes), while only four species are cultivated for textile industry [4,5]. Cultivated cotton species include two diploid A-genome species (2n = 2x = 26), *G. herbaceum* and *G. arboreum* known as Asiatic/African cottons and two tetraploid AD-genome species (2n = 4x = 52), *G. hirsutum* and *G. barbadense*, known as American cottons [6,7]. Results from previous studies have shown that the emergence of allotetraploid cotton species (AD genomes) was due to the cross-hybridization of diploid species belonging to the A and D genomes about 1.5 million years ago and their subsequent polyploidization [8,9]. The upland cotton species *G. hirsutum* (AD_1_) is of high productivity, has early maturity, and is easy to cultivate. Upland cotton crops are most widely distributed throughout the world, and its genetic diversity is extensively explored in breeding programs. 

The subspecies (ssp.) *purpurascens* belonging to the tetraploid *G. hirsutum* is a perennial cotton plant. It was first discovered in Asia, Africa, and South America in the 17th century [10]. This subspecies was classified a separate species of the *Gossypium* genus (*G. purpurascens*) according to Watt, 1907 [10]. However, Hutchinson and Stephens [11] believed that it should belong to the species *G. hirsutum*. Consequently, the ssp. *purpuracens* is left untouched by scientists among the seven geographical forms of the species *G. hirsutum*. To researchers who are interested in cotton improvement, this forgotten form is considered as a potential source of natural variation in plant’s adaptation to the environment and its tolerance to biotic and abiotic stresses. Because the reproductive stage of *G. purpurascens* does not continue the flowering under natural climatic conditions in temperate latitudes, it is only found in tropical or certain subtropical latitudes [12]. Plants belonging to this form grow naturally in the low latitudes for centuries without human intervention and have developed features such as sensitivity to photoperiods, adaptability to environmental conditions, and interesting plant architecture. The morphological differences and geographical isolation suggest that *G. purpurascens* would be a wild type of *G. hirsutum*.

Most wild and primitive accessions of *G. hirsutum* germplasm are sensitive to photoperiod, which makes it difficult to use such forms in the hybridization of cotton breeding. To solve this problem, existing classical breeding methods have been successfully used to photoperiodically introduce neutral genes in primitive forms of *G. hirsutum* to reduce sensitivity to the photoperiod in cotton. For example, photoperiodically neutral genes were transferred into 97 wild cotton accessions by backcrossing [13,14]. Because of this gene transfer, such accessions have become a valuable source of genetic diversity for the transfer of beneficial genes in cotton breeding material. An alternative method for obtaining photoperiodically neutral plants from wild species is the use of induced mutagenesis [15,16]. While these approaches have proven successful, they require lengthy and laborious research processes.

Plant species is often characterized with a pronounced amplitude of variation in botanical features and properties. As such, cotton species or even varieties can differ from each other in the shape of the bush, the shape of the leaves, the size of the flower corollas, branching, the shape of the bolls, etc. These morphological descriptors may be used to characterize the intraspecific polymorphism inherent in plants due to the manifestation of different forms of intraspecific variability [17]. Although exploiting the beneficial genetic variation of wild germplasm for traditional cotton breeding involves several complex processes, investigating radiation-induced day-neutral following is important in the scientific understanding of plant development and biochemical processes. In our previous research, we reported quantitative trait loci (QTLs), associated flowering-time, photoperiod insensitivity, and some other morphological and breeding important traits of wild cotton *Gossypium darwinii* Watt [16]. In this study, we report our morphological, cytological, and genomic analysis of mutagenesis-induced changes in cotton *Gossypium hirsutum* L. subsp. purpurascens.

## 2. Results

### 2.1. Phenotypic Characterization

**Figure 1:**
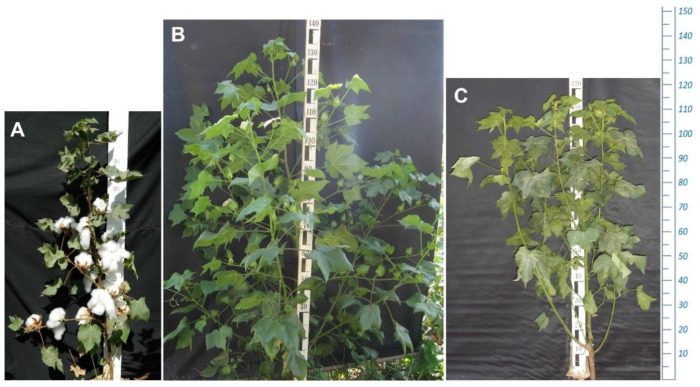
(**A**) ♀—Day-neutral mutant line (var. Kupaysin); (**B**) F_1_—hybrid of the first generation; (**C**) ♂—photoperiod-sensitive wild-type *G. hirsutum* subsp. *purpurascens* (var. El-Salvador).

**Figure 2:**
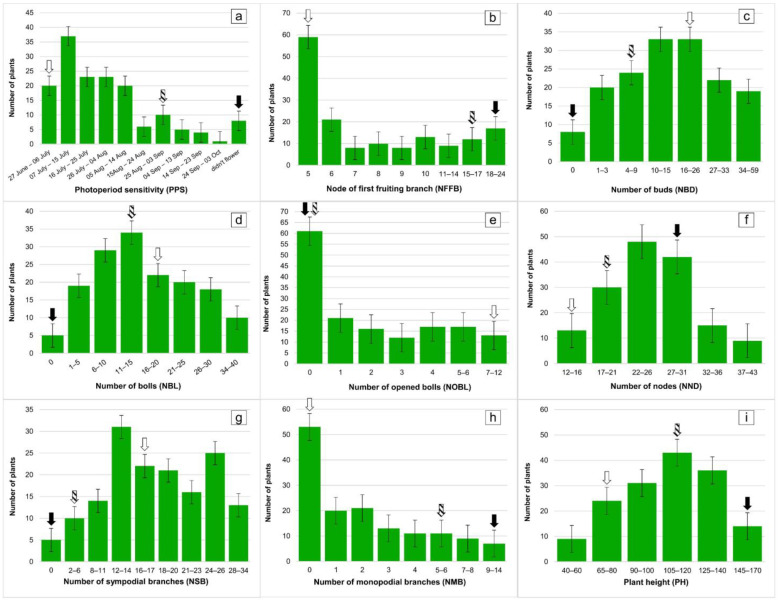
Histograms of the cotton traits related to flowering time and photoperiod sensitivity in the F_2_ progeny. (**a**) Photoperiod sensitivity (PPS); (**b**) node of first fruiting branch (NFFB); (**c**) number of buds (NBD); (**d**) number of bolls (NBL); (**e**) number of opened bolls (NOBL); (**f**) number of nodes (NND); (**g**) number of sympodial branches (NSB); (**h**) number of monopodial branches (NMB); (**i**) plant height (PH). The arrows show means for parental genotypes and F_1_ hybrid; white arrow—mutant line (Kupaysin); black arrow—wild type; arrow with patterned fill—F_1_ plant.

The F_1_ progeny was developed from a cross between the photosensitive wild-type “El Salvador” (*G. hirsutum* subsp. *purpurascens*) and its day-neutral mutant line, later registered in Uzbekistan as a promising cotton variety called “Kupaysin” (Figure 1). Based on the phenotypic observations of the F_2_ progeny along with their parental genotypes and F_1_ hybrids (5 plants each), morphological and economically valuable traits of cotton were identified (Table 1, Figure 2). An average value of traits in the F_1_ and F_2_ progeny was obtained with the following parameters. In the F_1_ hybrids, the following parameters were obtained: plant height (PH)—120 cm; the node of the first fruiting branch (NFFB)—at a node 17; number of monopodial branches (NMB)—5 pcs; number of sympodial branches (NSB)—5 pcs; number of nodes (NND)—21 pcs’ shape of bolls—ovoid; the number of bolls (NBL) is 13 and, of these, the number of opened bolls (NOBL) is 0; anthocyanin pigments are medium, and the bush shape is spreading.

In the F_2_ progeny, the following parameters were obtained: the plant height is 110 cm; the node of first fruiting branch average was 8.5. For the number of monopodial branches, four pieces were observed in 64% of plants; monopodial branches were not observed in the remaining 36% of plants. The number of sympodial branches was 17 pieces; the number of nodes was 25 pieces. For boll shape, out of 145 plants, 112 were egg-shaped, 23 were conical, 1 was spherical, and 9 of them did not form a box. Moreover, the number of flaps in the formed bolls was observed as 4–5 pieces. The number of bolls was 15/1 (total number of bolls/number of opened bolls); the branching type was unlimited apical growth; 1–3 types; anthocyanin pigment—intermediate. The degree of bush pubescence was as follows: strong—13%; medium—40%; weak (little)—47%. The bush shape was found: 12.3% of plants—compact; 87.7% of hybrids—open.

Table 1 shows that in the F_1_ progeny, the height of plant, the number of bolls, the anthocyanin stain, and the number of nodes were inheritable with spaces in relation to each of the two parents. As for the type of branching, the number of sympodial branches, and the shape of the bush, it was evident that the wild-type *G. hirsutum* ssp. *purpurascens* var. El-Salvador prevails. It was also noted that in the F_1_ progeny, NFFB also increased in comparison with the wild-type.

It was observed that the number of monopodial branches in the F_2_ progeny decreased in comparison with the wild-type (El-Salvador), as well as the F_1_ hybrids. The number of sympodial branches increased significantly compared to El-Salvador, as well as the F_1_ hybrids. The F_2_ progeny even showed better results than the Kupaysin variety. Plant height, the number of nodes, number of bolls, type of branching, the pubescence of the stem (strong, medium, and weak), bush form, shape of bolls, and the number of first fruiting branch were inheritable characters with an interval.

**Figure 3:**
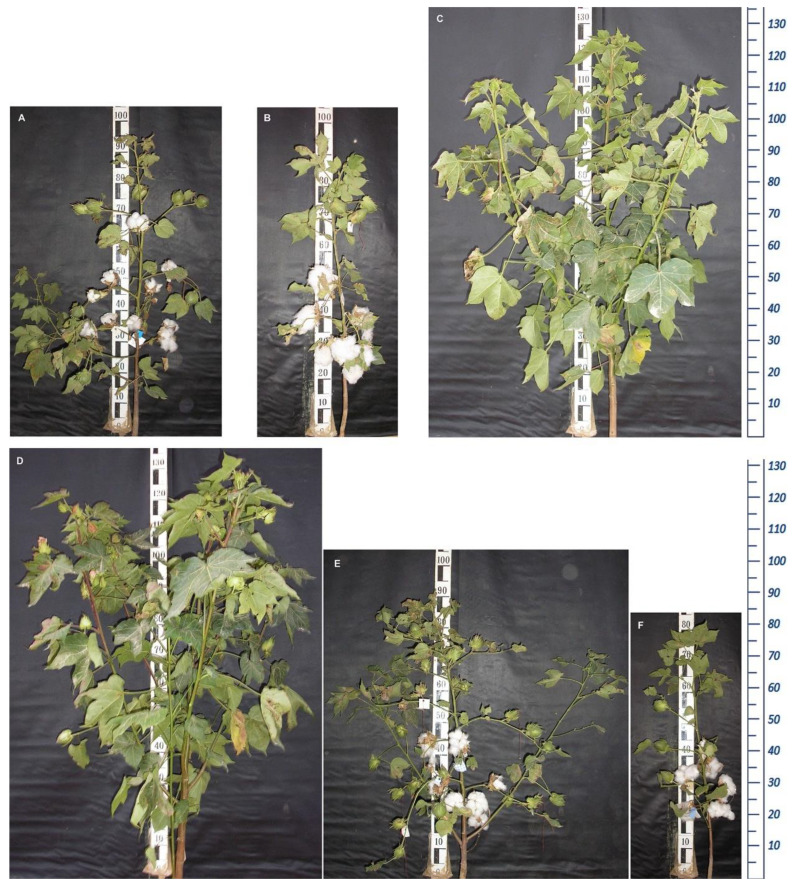
(**A**–**F**)—Cotton plant segregating genotypes of F_2_ progeny.

These characteristics observed in the F_1_ and F_2_ progenies show that Kupaysin dominated the number of bolls and the number of flaps in a boll. For the number of sympodial branches, anthocyanin spots, and the shape of a bush, El-Salvador appeared to dominate these traits. The average number of sympodial branches was higher in the F_2_ progeny’s percentage relative to the plant, and it was higher than that of parent plants and the F_1_ progeny. This allows one to select the cotton plants recombined with large numbers of sympodial branches among the F_2_ progeny. It was noted that there are genotypes among the F_2_ progeny that differ dramatically in their type of branching and early maturity (Figure 3).

Broad-sense heritability (*H*^2^) ranged from 0.07 to 0.90% for all studied traits (Table 2). All photoperiod sensitivity-related traits, such as flowering time, number of branches, and height of sympodia were found to have high heritability (0.40–0.90).

The F_2_ progeny was dramatically changed in comparison with the parents by plant height, number of nodes, number of first fruiting branch (NFFB), number of monopodial branches, number of sympodial branches, and number of bolls. In addition, high phenotypic polymorphisms were found between parental genotypes. Almost all observed flowering-related traits, such as photoperiod sensitivity, NFFB, number of sympodial branches, and plant height were estimated to have high levels of heritability (0.40–0.90). While it was identified, the traits—number of monopodial branches and number of opened bolls—had low levels of inheritability (0.18–0.07). Furthermore, donor genotypes could be identified among the F_2_ progeny with early maturity, and they were high-yielding for breeding programs.

### 2.2. Cytological Observation

**Figure 4:**
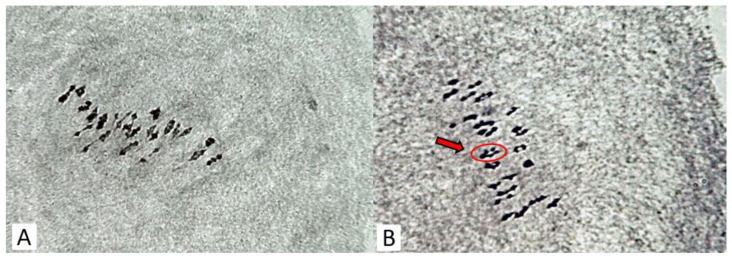
Chromosome conjugation at metaphase I of meiosis in cotton *G. hirsutum*: (**A**) photoperiod-sensitive wild-type *G. hirsutum* subsp. *purpurascens* (var. El-Salvador)—26 ^II^; (**B**) day-neutral mutant line (var. Kupaysin)—24 ^II^ + 1 ^IV^. Arrow indicates a quadrivalent.

The analysis of meiosis at the stage of metaphase I (MI) in ssp. *purpurascens* wild-type El-Salvador revealed the normal conjugation of chromosomes with the formation of 26 bivalents in pollen mother cells (PMCs) (Figure 4A). This indicated their cytogenetic homogeneity because no chromosome rearrangements were recorded at the MI stage of meiosis. In the mutant variant Kupaysin, however, they were characterized by the presence of quadrivalent chromosome associations in the studied PMCs with different frequencies (0.21 ± 0.11 and 0.14 ± 0.10 on average per cell, respectively) (Figure 4B). The quadrivalents had an open ring shape and an adjoining type of divergence of chromosomes from translocation complexes. The latter confirmed the existence of hidden structural chromosomal variability in the studied form of cotton.

**Figure 5:**
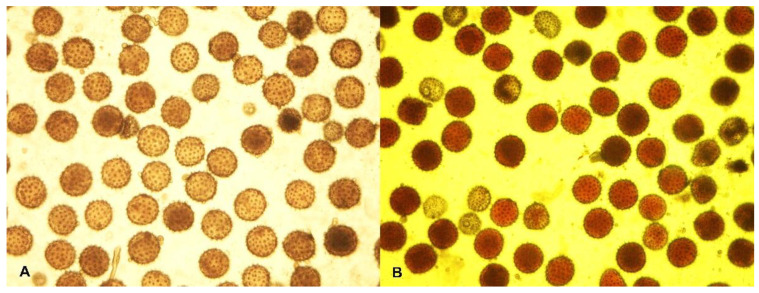
Pollen fertility: (**A**) photoperiod-sensitive wild-type *G. hirsutum* subsp. *purpurascens* (var. El-Salvador)—94.82 ± 0.52%; (**B**) day-neutral mutant line (var. Kupaysin)—88.51 ± 0.75.

**Figure 6:**
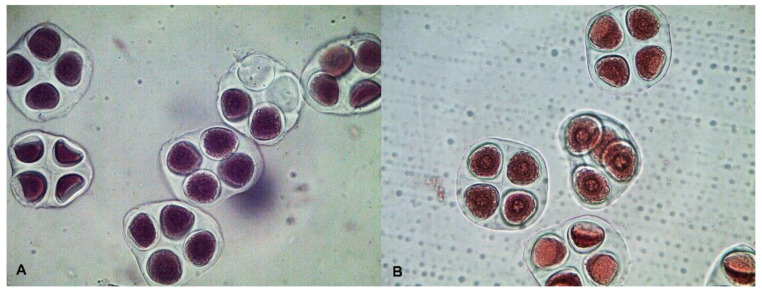
Meiotic index: (**A**) photoperiod-sensitive wild-type *G. hirsutum* subsp. *purpurascens* (var. El-Salvador)—94.15 ± 0.64%; (**B**) day-neutral mutant line (var. Kupaysin)—94.60 ± 0.59%.

A high percentage of viable pollen was observed in the original form ssp. *purpurascens* El-Salvador (94.82 ± 0.52). A slight decrease in pollen viability was observed in the mutant form Kupaysin (88.51 ± 0.75) (Figure 5). In sporadic analysis, both variants were found with a high meiotic index (94.15 ± 0.64; 94.60 ± 0.59%) (Figure 6).

### 2.3. SSR Genotyping

**Figure 7:**
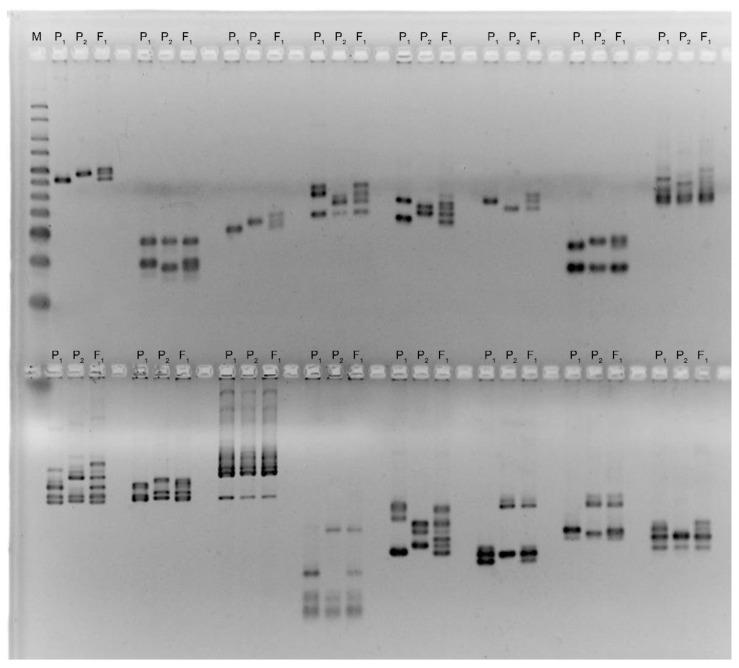
PCR analysis of parent genotypes and F_1_ hybrids using GH primers. P_1_—photoperiod-sensitive wild-type *G. hirsutum* subsp. *purpurascens* (var. El-Salvador); P_2_—day-neutral mutant line (var. Kupaysin); F_1_—first-generation hybrid.

The genetic diversity between parental samples was observed with public cotton simple sequence repeat (SSR) markers [18]. The DNA polymorphism between parental genotypes was determined using CH, TMB, BNL, and JESPR markers (Figure 7). The genotyping of the F_2_ population was performed using polymorphic SSR markers between the two parents in this study. To determine the polymorphism, parental genotypes were surveyed by PCR using 336 SSR markers. The PCR analysis showed that 69 SSRs were found to be polymorphic among 336 SSRs between the parental genotypes. Polymorphism was observed in 12 samples with 48 GH markers, 26 samples with 144 TMB markers, 21 samples with 96 BNL markers, and 10 samples with 48 JESPR markers. The group of primers that detected the most polymorphisms between the parent samples was the GH collection.

In the F_2_ population, high levels of polymorphism were identified between the parental genotypes for plant height, the number of first fruiting branch, the number of monopodial branches, and the number of sympodial branches. Such SSR polymorphisms facilitates mapping the genetically linked QTLs with the morphological and economically valuable traits. The molecular marker technology was a useful tool for studing the complex quantitatively inherited traits [19].

### 2.4. Genetic Linkage Groups

Genetic linkage groups (LGs) were constructed, and they mapped 63 SSR markers that were the polymorphic (Table 3) in the F_2_ population and the remaining six SSRs were unmapped. Nine LGs covered a total of 857.2 cM with an average distance of 13.6 cM between two markers.

The nine LGs represented eight cotton chromosomes based on the previously published genetic maps (Table 4). Each LG belongs to one chromosome except for chromosome 23, which has two LGs. Linkage group 2 (LG02) was the largest group that consists of 15 SSR markers. The genetic distance of LG02 was 144.8 cM, and the average distance between two loci was 9.7 cM. LG04 (At-11), LG05 (At-13), and LG09 (Dt-09b) consisted of only five, six, and four markers, respectively. The smallest linkage group was LG04 (At-11) with the shortest genetic distance (8.1 cM). The locations of these markers on cotton chromosomes were determined upon genetic studies conducted using 63 SSR markers and published by mapping data [18].

### 2.5. QTL Mapping

**Figure 8:**
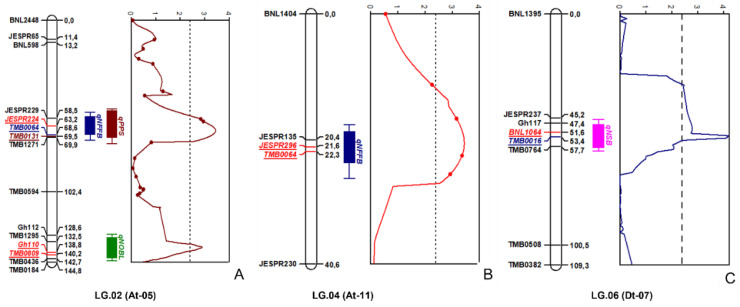
QTL maps of the F_2_ population showing the location of QTLs for photoperiodic sensitivity. (**A**) LG.02 (At-05), (**B**) LG.04 (At-11), and (**C**) LG.02 (At-05). The QTLs: *qPPS***—**photoperiod sensitivity; *qNFFB*—node of first fruiting branch; *qNOBL*—number of opened bolls; *qNSB*—number of sympodial branches. The map distance (centimorgan) is shown in comma decimal as direct output of the MapChart program.

Table 5 shows the association between morphological and economically important traits of cotton, including “flowering time” and SSR marker loci. The genetic linkage maps and phenotypic data were used to identify QTLs for flowering time and other related traits based on the F_2_ mapping population. Five QTLs were identified and associated with four traits related to flowering time and morphological features by composite interval mapping (CIM) with an LOD ≥5 (*p* ≤ 0.05 after 1000 times permutation test; Table 5) that explained at least 18% of the trait’s variations. Five QTLs for qNFFB, qNOBL, qPPS, and qNSB traits were located on chromosomes At-05, At-11, and Dt-07, respectively (Figure 8).

The chromosomal position of these QTL-associated markers was also suggested according to the published literature on cotton (Table 5).

### 2.6. Candidate Gene Annotation

When genotyping the wild-type El-Salvador and mutant Kupaysin cultivars with 336 SSR markers, amplification results involving 69 pairs of primers showed that there was a genetic mismatch between them. Therefore, in silico PCR was performed using UGENE 42.0 bioinformatics software package. Out of 69 markers used for in silico PCR analysis, only 59 detected a total of 75 virtual amplicon products, while 10 did not produce PCR products. Of these 75 PCR amplicon products, 63 were in silico amplified in 20 chromosomes, except for 6 chromosomes: At-02, At-03, At-04, At-08, Dt-03, and Dt-13. In contrast, the remaining 12 virtual PCR amplicons were found in unannotated regions of the chromosomes (or scaffolds) (Table S1). The position of in silico SSR amplicons was identified on chromosomes or scaffolds of the *G. hirsutum* genome according to Yu et al. 2021 [18]. The results of the in silico PCR showed that three amplicons were synthesized with each of four markers, two amplicons with each of the eight markers, and one amplicon each with the remaining forty-seven markers.

Most amplicons were observed on cotton chromosomes At-09 and Dt-01 (7 markers in each chromosome). Six PCR products were amplified on cotton chromosome Dt-09 and five each on chromosome At-06 and Dt-10. Four marker plots were observed on chromosome At-10 and in scaffold 445.1. The least virtual PCR product corresponded to the cotton chromosomes At-05, At-12, Dt-02, Dt-07, and Dt-08, with only one amplicon observed on these chromosomes. In the analysis of selected SSR markers, virtual PCR products were detected from the cotton chromosomes At-02, 3 At-03, At-04, At-08, Dt-03, and Dt-13.

Six QTLs, related to flowering time, were selected to identify candidate genes via a BLAST search. In silico PCR analysis of Upland cotton genome sequence with these SSR markers produced 75 virtual amplicons (Table S1). The in silico SSR amplicons were located on chromosomes or scaffolds of the *G. hirsutum* genome [25]. Nineteen candidate genes/proteins with known functions in cotton, as well as in other organisms, were indicated through a comparative analysis of the amino acid sequences of a total of 94 putative genes and their transcripts found in the genomic regions of virtual amplicons using the protein BLAST algorithm (the pairwise alignment of protein/protein sequences) (Table S2).

## 3. Discussion

Wild and primitive accessions of cotton germplasm are mostly sensitive to the photoperiod, limiting the use of the genetic diversity essential for the improvement of cotton crop. In this study, we analyzed a wild-type *Gossypium hirsutum* perennial subsp. *purpurascens* var. El-Salvador that is known for its adaptation to the adverse environments and its tolerance to biotic and abiotic stresses. Upon the treatment with gamma radiation, the mutant cotton var. Kupaysin exhibited day-neutrality in flowering or insensitivity to the photoperiod [16]. The radiation-mediated mutagenesis induced many morphological, cytological, and genomic changes. Some of the changes related to the flowering are highly inheritable, indicating that they were transmitted to the progeny of the mutant cotton (Table 2).

Cytological data suggest that in PMCs of wild-type El-Salvador, the conjugation of chromosomes was homogeneous with the formation of 26 bivalents. In the mutant variant Kupaysin, however, they were characterized by the presence of quadrivalent chromosome associations in the studied PMCs with different frequencies (Figure 4). The relationships of such cytogenetic abnormalities to photoperiod insensitivity and other related traits remain to be further investigated.

QTL mapping analyses were conducted by using SSR markers and an F_2_ population derived from a cross between the wild-type cotton El-Salvador and its mutant Kupaysin. The five QTLs identified in the study were associated with the four traits related to flowering time, node of first fruiting branch, number of opened bolls, and number of sympodial branches (Figure 8). The close relationship between the mapping parents (the wild-type and its mutant) resulted in the low percentage of SSR markers that were polymorphic for genotyping the F_2_ progeny and establishing linkage groups. Thus, other observed traits of mutagenesis such as plant height, number of monopodial branches, number of nodes, and number of total bolls would require additional polymorphic markers to detect QTLs.

The in silico analysis of the genomic regions of QTLs associated with flowering related traits produced 75 virtual amplicons. Further studies are necessary, such as real-time PCR and actual cloning of the nineteen candidate genes/proteins that were indicated with protein BLAST. For example, the corresponding amino acid sequence of the TMB0064 marker region had genes encoding aspartic proteinase 36-like. In plants, aspartic proteases, which belong to one of the four major mechanistic classes of proteolytic enzymes with conserved motifs Asp-Thr/Ser-Gly (DT/SG), display aspartic proteolytic activity [26]. Moreover, together with A39, aspartic proteinase 36 contributes to pollen and ovule development, including the apical cell wall constitution of the growing pollen tubes.

Similarly, the region of the genome, where the BNL1064 SSR marker is located, is likely to contain genes encoding Aspartic proteinase 36-like proteins [26]. Moreover, in this genome region, we identified genes coding proteins, such as phosphoserine phosphatase, chloroplast-like, polyadenylate-binding protein 2-like, putative pentatricopeptide repeat-containing protein At1g26500, and spindle pole body component 110-like. According to Jiang et al. (2015) [27], in Arabidopsis thaliana, pentatricopeptide repeat protein SOAR1 that functions to negatively regulate abscisic acid (ABA) signaling in seed germination and post-germination growth was a crucial and positive regulator of plant responses to abiotic stresses.

Coronatine-insensitive protein 1 is identified on marker region Gh110 belonging to the chromosome Dt-10, which was involved in the dorsoventral asymmetry of flowers [28]. In the genomic region where flanking marker TMB0809 was located, genes encoding pentatricopeptide repeat proteins (PPR) were found. The *GhImA* gene-encoded PPR protein attends mitochondrial nad7 splicing, influences respiratory metabolisms, and controls cotton fiber development via ATP supplies and the ROS balance [29]. SSR marker TMB0131 was associated with photoperiod sensitivity, and within the proximity of this genomic locus, there was a candidate gene called Aldehyde dehydrogenase family 3 member F1. This gene was reported to function in the development of cotyledons and leaves [30].

Of particular interest to this study were the genes found in the TMB0016 microsatellite marker region. Thus, this genomic region likely contained the Homeobox protein LUMINIDEPENDENS, which regulates flowering time in the autonomous flowering pathway by repressing FLOWERING LOCUS C expression [31]. In addition, protein indeterminate-domain 2-like, which plays a redundant role with IDD14 in directing leaf and floral organ morphogenesis, has been found here [32].

Future studies may confirm the functions of these candidate genes in the effects of cotton-flowering-related traits. The DNA markers and candidate genes associated with these traits can facilitate cotton molecular breeding programs to introduce new genetic variations from wild cotton [19].

## 4. Materials and Methods

### 4.1. Plant Materials

As described in the previous studies [15,33,34], more than 200 mutant lines of wild and primitive representatives of diploid and tetraploid cotton species were created that induced photoperiod insensitivities via radiation-mediated mutagenesis. Among these mutants, cotton cultivars including Kupaysin with improved economically valuable traits were developed after the exposure of the photoperiod sensitive wild type of *G. hirsutum* subsp. *purpurascens* var. El Salvador with γ-rays of ^32^P radioactive isotopes [16]. Field experiments were conducted in 2006–2008 in the nursery at the Institute of Genetics and Plant Experimental Biology (IGPEB), Academy of Sciences of Uzbekistan, located in Qibray district, Tashkent (a temperate region of 41.38 latitude and 69.46 longitude). The photoperiod sensitive wild-type El Salvador belonging to the *G. hirsutum* subspecies *purpurascens* and its day-neutral mutant line (Kupaysin) was chosen to create a specific experimental F_2_ population for photoperiod sensitivity by crossing them with each other. The seeds of parental genotypes were taken from the cotton germplasm collection at IGPEB.

### 4.2. Phenotypic Observation

In 2006, the mutant line Kupaysin and its wild-type El-Salvador were grown at field stations (naturally long-day conditions) and evaluated for their photoperiod sensitivity and agronomic traits. For crossing, special attention was given to the nature of the sensitivity of the day length of the wild-type El-Salvador. Due to the photoperiod sensitivity, the wild-type was planted simultaneously in the greenhouse under the control of an artificially short day (8 h of light and 16 h of darkness). It was the only way to achieve the flowering of El-Salvador and to cross with its neutral-day mutant line Kupaysin. F_1_ hybrids, as well as their parental lines, were grown and phenotypically observed during the 2007 season. To obtain the F_2_ progeny of the experimental population, the F_1_ hybrids were self-pollinated. At the end of April 2008, 157 F_2_ segregating progenies, as well as parent genotypes and their F_1_ hybrids (five plants from each), were planted at a plant-to-plant spacing of 90 cm × 20 cm in a natural day condition. To study photoperiod sensitivities, all F_2_ individuals were phenotypically measured for nine traits related to flowering and photoperiod-sensitivity in cotton, such as (i) photoperiod sensitivity (PPS); (ii) node of first fruiting branch (NFFB); (iii) number of buds (NBD); (iv) number of bolls (NBL); (v) number of opened bolls (NOBL); (vi) number of nodes (NND); (vii) number of sympodial branches (NSB); (viii) a number of monopodial branches (NMB); and (ix) plant height (PH)**.**

### 4.3. Heritability Calculation

The broad-sense heritability (*H*^2^) for each trait was estimated according to Singh et al. (1993) [35] using the following formula.

*H*^2^ = *V_g_*/*V_p_*,

where *V_g_* is the genetic variance and *V_p_* is phenotypic variance.

The phenotypic variance (*V_p_*) and the genotypic variance (*V_g_*) were found by the following formulas:

*V_p_* = *V_g_* + *V_e_* and *V_g_* = *V_p_* − *V_e_*.

Here, *V_e_* is the environmental variance. The environmental variance (*V_e_*) was estimated using formula

*V_e_* = (*VP*1 + *VP*2)/2,

where *VP*1 and *VP*2 were the variances of the respective parental genotypes.

### 4.4. Cytological Analysis

The analysis of meiosis, at the stage of metaphase-I (MI) in pollen mother cells (PMC), as well as the analysis of tetrads, was conducted according to Sanamyan et al. 2022 [36]. In the morning, 2–4 mm cotton buds were fixed in an alcohol–acetic mixture at a ratio of 3:7. After a preliminary analysis of the recorded bud material by stages of fiber development and the selection of buds at the stages of metaphase-I and tetrads, PMCs were stained with iron-acetocarmine. Metaphases of the first division of meiosis were analyzed on temporarily crushed preparations under a light microscope and the nature of chromosome conjugation was taken into the account. For the analysis of sporades, several buds from each variant were analyzed and the meiotic index (Mi) was calculated as the percentage of normal tetrads over total sporades. To analyze the pollen fertility in the morning on the day of flowering, opened flowers were collected, temporary acetocarmine preparations were performed, which were placed in Petri dishes and incubated in the refrigerator for a day to better stain pollen grains. Approximately 10 fields of view were analyzed per sample. A Leica CM E microscope with a Leica EC3 camera was used to conduct cytological studies.

### 4.5. DNA Isolation and SSR Analysis

The genomic DNA of experimental cotton plants in this study was isolated with the cetyltrimethylammonium bromide (CTAB) method [37]. The DNA concentration was determined by visually comparing the DNA of the lambda (λ) phage with the exact concentration (25 ng/µL) in 0.8% agarose gel. DNA samples were diluted to a working concentration of 25 ng/µL. A total of 336 simple sequence repeat (SSR) primer pairs were selected from the cotton marker collection https://www.cottongen.org (accessed its progenitor database CottonDB on 10 November 2008) for molecular research [25]. PCR assays and SSR genotyping were conducted as described previously [18].

### 4.6. Linkage Mapping and QTL Analysis

Genotyping data were processed using the JoinMap 3.0 mapping program in accordance with the manual instructions. All linkage groups were developed at a minimum LOD value (LOD-score: logarithm of odds) of 4.0 and a maximChart 2.2 software [38] was used to draw the linkage groups, which contain SSR loci in the cotton chromosomes. Linkage groups were assigned to the respective chromosomes based on published data on the linkage of identical markers on a high-density cotton genetic map [18,39]. Quantitative traits locus (QTL) analyses in the F_2_ progeny were performed using QTL Cartographer v2.5 [40] using Kosambi [41] as a mapping function, applying the algorithm of composite interval mapping (CIM). The threshold LOD levels were identified using QTL Cartographer and Qgene [42] at the 95% significance level after 1000 times permutation test. An association between phenotypic and genotypic data was identified using a simple regression analysis. The chi-square value (χ^2^) of segregating markers, as well as regression analysis of each marker, was performed using the QGene 3.06 software. The position of QTL loci in linkage groups was determined using the composite interval mapping (CIM) algorithm. The significance of the relationship between the identified loci and polymorphism for a particular trait was assessed on the basis of the LOD threshold value. An individual QTL analysis was performed for each trait using WinQTL cartographer [39]. The nomenclature of each QTL includes “q” followed by an abbreviation for the name of the trait, chromosome, or linkage group, and a serial number to distinguish between different QTLs of the same trait on the same chromosome.

### 4.7. In Silico Analysis

In silico PCR analysis was performed in the UGENE 42.0 bioinformatics software package [43], using the appropriate primer pairs of the selected SSR markers and the DNA sequence of the complete genome of *G. hirsutum* [44]. The primer sequences of cotton SSR markers were obtained from the CottonGen database (https://www.cottongen.org/, accessed on 4 April 2022). To detect and predict the candidate genes, we used web application AUGUSTUS 3.1.0 and the National Center for the Biotechnology Information (NCBI) Basic Local Alignment Search Tool (BLAST) database. The search for candidate genes/proteins in the NCBI database was performed along the genomic regions of 100,000 bp located above and below each marker locus in the DNA sequence using BLAST analysis. This was followed by a thorough review of the literature to elucidate the function of the discovered genes, as well as an analysis of the metabolic pathways involving these genes using the latest version of the Kyoto Encyclopedia of Genes and Genomes (KEGG) database PATHWAY 103.0, released 1 July 2022 (http://www.genome.jp/kegg/pathway.html, accessed on 12 July 2022) [45].

## 5. Conclusions

Several morphological and agronomical traits of the mutant cultivar Kupaysin were analyzed upon the radiation-mediated mutagenesis of El-Salvador, the wild-type *G. hirsutum* subsp. *purpurascens*. Plant height, number of first fruiting branch, monopodial and sympodial branching, flowering time duration, sensitivity to photoperiod, and characteristics such as prematurity changed dramatically relative to the original wild-type. Cytological changes were observed in cotton chromosomes upon mutagenesis. Genomic regions containing the polymorphic SSR markers were studied with in silico PCR assays. QTLs associated with flowering-related traits were identified, and candidate genes responsible for these traits were suggested in the *G. hirsutum* genome. The future functional analysis of the candidate genes identified from this study would facilitate the enhanced utilization of novel genetic diversity for molecular cotton breeding programs.

## Figures and Tables

**Table 1:** Average values of morphological and agronomical traits of the parents, F_1_ hybrid, and F_2_ progeny.

Traits	Mutant Line—Kupaysin	Wild Type (El-Salvador)	F_1_ Generation	F_2_ Generation				
				Min	Max	Mean	SD *	SE **
Plant height (cm)	80	175	120	40	170	109.33	26.96	2.15
Number of monopodial branches	0	7	5	1	14	3.93	3.16	0.33
Number of sympodial branches	15	5	5	2	34	17.70	6.99	0.58
Number of nodes	18	26	21	12	43	25.06	5.58	0.46
Shape of bolls	ovoid	ovoid	ovoid	23-cone-oval, 1-round, 112-ovoid				
Number of bolls per plant	15	0	13	1	40	15.50	8.90	0.74
Number of opened bolls per plant	7	0	0	0	12	2.17	2.63	0.22
Anthocyanin	Strong	Strong	Strong	Strong				
Bush shape	Compact	Spreading	Spreading	Compact—12.3%, Spreading—87.7%				
Node of first fruiting branch	4	13–14	17	5	24	8.48	4.88	0.41

* SD: standard deviation; ** SE: standard error.

**Table 2:** Genetic variance and estimated broad-sense heritability (*H*^2^) of morphological and agronomical traits of the F_2_ progeny, including their parental genotypes.

	PPS	PH	NFFB	NMB	NSB	NBL	NOBL
P_1_	0.00	8.41	2.56	2.45	2.11	0.00	0.00
P_2_	4.50	8.23	0.71	1.12	2.32	2.61	2.72
F_2_	22.80	38.41	4.85	3.68	8.53	11.08	1.47
V_g_	20.55	25.89	1.94	0.67	5.26	9.78	0.11
*H* ^2^	0.90	0.67	0.40	0.18	0.62	0.88	0.07

P_1_—photoperiod-sensitive wild-type *G. hirsutum* subsp. *purpurascens* (var. El-Salvador); P_2_—Day-neutral mutant line (var. Kupaysin); F_2_—second generation population; V_g_—genotypic effect; *H*^2^—broad-sense heritability; PPS—photoperiod sensitivity; PH—plant height; NFFB—node of first fruiting branch; NMB—number of monopodial branches; NSB—number of sympodial branches; NBL—number of bolls; NOBL—number of opened bolls.

**Table 3:** Sets of mapped and unmapped SSR markers.

SSR Marker Collection	Number of Mapped Markers	Number of Unmapped Markers
BNL SSR	19	2
GH SSR	11	1
JESPR SSR	9	1
TMB SSR	24	2
Total:	63	6

**Table 4:** List of genetic linkage groups constructed with the F_2_ progeny.

Linkage Groups	Chromosome	Number of Mapped Loci	Total Map Length (cM)	Mean Map Distance/Marker
LG01	At-03	8	121.7	15.2
LG02	At-05	15	144.8	9.7
LG03	At-07	4	88.3	22.1
LG04	At-11	5	40.6	8.1
LG05	At-12	6	59.9	10.0
LG06	Dt-07	8	109.3	13.7
LG07	Dt-05	12	129.8	10.8
LG08	Dt-09a	7	108.6	15.5
LG09	Dt-09b	4	54.2	13.6
Total:		63	857.2	13.6

**Table 5:** Genomic distribution of SSR markers and QTLs related to flowering time and morphological traits.

#	QTL	Linkage Group	Chromosomal Location	Linked Markers	Position (cM)	LOD	Literature Reports	
							Association of Markers with Other Traits	Reference
1	*qNFFB*	LG02	At-05	JESPR224_175-TMB0064_180	60.20–69.72	3.64	Fiber micronaire (Dt-06);	[20]
							Fiber strength (Dt-06)	
							FOV race 4 resistances (Dt-06)	[21]
							Flowering time (At-11)	[19]
							Fiber length (At-11)	[22]
2	*qNOBL*	LG02	At-05	GH110_130-TMB0809_205	135.90–141.20	3.24	Total fruit nodes;	[23]
							Fruit branch angle (Dt-10).	
3	*qPPS*	LG02	At-05	JESPR224_175-TMB0131_240	58.90–69.91	3.64	Fiber micronaire (Dt-06);	[20]
							Fiber strength (Dt-06)	
							FOV race 4 resistances (Dt-06)	[21]
4	*qNFFB*	LG04	At-11	JESPR296_135-TMB0064_200	21.20–22.40	3.71	Fiber micronaire (Dt-06)	[20]
							Flowering time (At-11)	[16]
							Fiber length (At-11)	[22]
5	*qNSB*	LG06	Dt-07	BNL1064_150-TMB0016_250	48.30–56.40	4.2	Fiber uniformity (Dt-06).	[24]

The QTLs; *qPPS***—**photoperiod sensitivity; *qNFFB*—node of first fruiting branch; *qNOBL*—number of opened bolls; *qNSB*—number of sympodial branches.

## Data Availability

Not applicable.

## Supplementary Materials

The following supporting information can be downloaded at: https://www.mdpi.com/article/10.3390/plants11223012/s1. Table S1: List of virtually amplified SSR markers; Table S2: List of candidate genes that were predicted with QTL-associated markers.
