# Supplementary material for: Genetic Analysis of Mutagenesis That Induces the Photoperiod Insensitivity of Wild Cotton Gossypium hirsutum Subsp. purpurascens
Source: Plants (Basel). 2022 Nov 8;11(22):3012. doi: 10.3390/plants11223012 (PMC9698681; doi:10.3390/plants11223012)
Supplement: Supplementary file 1 [file plants-11-03012-s001.zip › plants-1952316-supplementary.pdf]

Table S1. List of virtually amplified SSR markers.

№	Name of amplified markers	Amplicon copy	Positions of polymorphic markers on the genetic map (Yu et al., 2021)	Physical positions of markers
1	BNL358	1	Dt-04	Dt-12
2	BNL530	1	At-04	Dt-12
3	BNL542	1	At-05	At-09
4	BNL598	2	At-12	Dt-08, scaffold1554.1
5	BNL686	1	At-09, Dt-09	scaffold559.1
6	BNL830	1	Dt-01	Dt-02
7	BNL946	1	Dt-10	Dt-11
8	BNL1064	1	At-06	At-10
9	BNL1317	3	At-09, Dt-09	At-06, Dt-06, scaffold4972.1
10	BNL1395	1	Dt-07	Dt-01
11	BNL1404	1	At-11, Dt-11	At-07
12	BNL1421	1	At-13	At-13
13	BNL1495	1	At-13	At-13
14	BNL1521	1	Dt-08	scaffold293.1
15	BNL1604	1	At-07, Dt-07	Dt-01
16	BNL1672	1	At-09, Dt-09	Dt-06
17	BNL1694	2	At-07, Dt-07	Dt-01
18	BNL2448	1	At-05	At-09
19	BNL2496A	2	Dt-02	At-05, Dt-01, scaffold290.1
20	GH032	1	At-06	scaffold445.1
21	GH039	1	At-06	scaffold445.1
22	GH056	1	Dt-07	Dt-01
23	GH083	1	At-05	At-09
24	GH110*	1	Dt-10	scaffold1251.1
25	GH112	1	At-09	At-06
26	GH171	1	Dt-08	Dt-04
27	GH211	1	At-05	At-09
28	GH215	1	At-13	At-13
29	GH224	1	Dt-06	Dt-10
30	GH247	3	At-09	At-06, Dt-06, scaffold4972.1
31	TMB0016*	1	Dt-08	Dt-04
32	TMB0064*	1	At-11	At-01
33	TMB0131*	3	At-05	At-09, At-09, Dt-09
34	TMB0154	1	At-06	scaffold445.1
35	TMB0161	1	At-10	Dt-11
36	TMB0184	1	At-09	At-06
37	TMB0382	1	Dt-09	Dt-05
38	TMB0419	2		Dt-07, At-07
39	TMB0426	1	At-11	At-01

40	TMB0429	1	Dt-08	Dt-10
41	TMB0436	2	At-06, Dt-06	At-11, Dt-10
42	TMB0482	1	At-13	scaffold2678.1
43	TMB0508	2	Dt-06	At-10, Dt-10
44	TMB0561	1	At-07, Dt-07	Dt-01
45	TMB0594	1	Dt-02	Dt-05
46	TMB0809*	1	At-4	At-12
47	TMB0812	2	At-10, Dt-10	Dt-09, Dt-11
48	TMB0853	1	At-06	scaffold445.1
49	TMB1271	1	Dt-07	Dt-01
50	TMB1277	1	At-06	At-10
51	TMB1288	1	At-10	At-11
52	TMB1295	2	Dt-05	Dt-09, Dt-09
53	JESPR65	1	Dt-04, At-05, At-07	At-09
54	JESPR135	1	At-11, Dt-11	At-07
55	JESPR204	3	Dt-05, At-13, Dt-13	At-10, At-06, Dt-09
56	JESPR229	1	Dt-06	Dt-10
57	JESPR230	1	At-09, Dt-04, Dt-05	Dt-12
58	JESPR236	1	Dt-05	Dt-09
59	JESPR296*	1	At-11	At-01
	**Total:**	**75**		

*Flanking QTL markers, associated with flowering time-related traits

Table S2. List of candidate genes that were predicted with QTL-associated markers.

No.	Predicted gene	Marker	Associated trait	Traits in literature	AUGUSTUS gene number	E value	Gene function
1	Aspartic proteinase 36-like	TMB0064_180	Node of first fruiting branch (*NFFB)*	Fiber strength, FOV race 4 resistances, Flowering time, Fiber length, Fiber micronaire	10	0E+00	Displays aspartic proteolytic activity (PubMed:27872247).<br>Together with A39, contributes to pollen and ovule development, including the apical cell wall constitution of the growing pollen tubes (PubMed:27872247)
2	1-aminocyclopropane-1-carboxylate oxidase homolog 1-like				3	2E-11	Enzyme involved in the ethylene biosynthesis. May promote stem elongation by maximizing the extensibility cells, possibly by activating ethylene biosynthesis, in response to very-long-chain fatty acids (VLCFAs C20:0 to C30:0).
3	Coronatine-insensitive protein 1	Gh110_130	Number of opened bolls (NOOB)	Number of sympodia	4	0E+00	Required for jasmonate-regulated plant fertility and defense processes, and for coronatine and/or other elicitors perceptions/responses. Seems to not be required for meiosis. Required for the regulation of some genes induced by wounding, but not for all. Component of SCF(COI1) E3 ubiquitin ligase complexes, which may mediate the ubiquitination and subsequent proteasomal degradation of target proteins (probably including the ribulose bisphosphate carboxylase small chain 1B RBCS-1B and the histone deacetylase HDA6). These SCF complexes play crucial roles in regulating response to jasmonate, and their interactions with the COP9 signalosome (CSN) appear to be important for their activity. Interacts with TIFY10A and inositol pentakisphosphate to form a high-affinity jasmonates coreceptor. Involved in the regulation of plant gene expression during plant-pathogen interactions with Pseudomonas syringae and Alternaria brassicicola.
4	Transcription factor DIVARICATA-like				5, 6	0E+00	Involved in the dorsovental asymmetry of flowers. Promotes ventral identity.

5	Carboxypeptidase A2-like (LOC108452605), mRNA	TMB0809_205	*Number of opened bolls (NOOB)*	Number of sympodia	8	0E+00	Transfers mannose from GDP-mannose to dolichol monophosphate to form dolichol phosphate mannose (Dol-P-Man) which is the mannosyl donor in pathways leading to N-glycosylation, glycosyl phosphatidylinositol membrane anchoring, and O-mannosylation of proteins; catalytic subunit of the dolichol-phosphate mannose (DPM) synthase complex. Plays a role in plant development and physiology, sensitivity to ammonium stress and endoplasmic reticulum stress response.
6	Pentatricopeptide repeat-containing protein At4g21065-like				3	0E+00	May function as a transcriptional regulator essential for early embryogenesis.
7	Conserved oligomeric Golgi complex subunit 1-like				9	1E-111	Involved in pollen tube growth by modulating Golgi morphology and vesicle trafficking homeostasis leading to the deposition of cell wall components and proteins at the pollen tube tip (PubMed:27448097). Required for sporophytic development (PubMed:27448097)
8	Aaldehyde dehydrogenase family 3 member F1	TMB0131_240	Photoperiod sensitivity (PPS)	Fiber strength, FOV race 4 resistances	4	5E-16	Required for the maintenance of the shoot apical meristem (SAM) structure and subsequent adaxial-abaxial axis-dependent development of cotyledons and leaves (PubMed:21690177, PubMed:25840087).
9	Pentatricopeptide repeat-containing protein At5g50990				4	8E-14	May function as a transcriptional regulator essential for early embryogenesis.
10	DEAD-box ATP-dependent RNA helicase 16				6	0E+00	Involved in leaf polarity establishment by functioning cooperatively with AS2 to repress abaxial genes ARF3, ARF4, KAN1, KAN2, YAB1 and YAB5, and the knox homeobox genes KNAT1, KNAT2, KNAT6, and STM to promote adaxial development in leaf primordia at shoot apical meristems at high temperatures. Involved in the processing of pre-rRNA intermediates at high temperatures.
11	Aspartic proteinase 36	BNL1064_150	Number of sympodial branches (NSB)	Fiber uniformity	2	0E+00	Displays aspartic proteolytic activity (PubMed:27872247).<br>Together with A39, contributes to pollen and ovule development, including the apical cell wall constitution of the growing pollen tubes (PubMed:27872247).

12	Phosphoserine phosphatase, chloroplastic-like				5	0E+00	Catalyzes the last step in the plastidial phosphorylated pathway of serine biosynthesis (PPSB). The reaction mechanism proceeds via the formation of a phosphoryl-enzyme intermediates. Required for embryo, pollen and root development. May be required preferentially for serine biosynthesis in non-photosynthetic tissues.
13	Polyadenylate-binding protein 2-like				6	0E+00	Promotes polyploidy in dark-grown seedlings. Regulates the endocycle leading to hypocotyl elongation.
14	Putative pentatricopeptide repeat-containing protein At1g26500				6	0E+00	Involved during embryo development.
15	Spindle pole body component 110-like				4	4E-93	Component of the spindle pole body (SPB) required for the proper execution of spindle pole body (SPB) duplication. Potential role in cross-linking filaments or anchoring other molecules. It is essential for growth (By similarity).
16	Cullin-1-like	TMB0016_250	Number of sympodial branches (NSB)	Fiber uniformity	5 t1, 5 t2	0E+00	Involved in ubiquitination and subsequent proteasomal degradation of target proteins. Regulator of mitotic processes which plays a role during gametogenesis and embryogenesis. Together with SKP1, RBX1 and a F-box protein, it forms a SCF complex. The functional specificity of this complex depends of the type of F-box protein. SCF(UFO) is implicated in floral organ development. SCF(TIR1) is involved in auxin signaling pathway. SCF(COI1) regulates responses to jasmonates. SCF(EID1) and SCF(AFR) are implicated in phytochrome A light signaling. SCF(ADO1/ZTL), SCF(ADO2/LKP2), SCF(ADO3/FKF1) are related to the circadian clock. SCF(ORE9) seems to be involved in senescence. SCF(EBF1/EBF2) may regulate ethylene signaling.
17	Homeobox protein LUMINIDEPENDENS				6	0E+00	May play a role in the regulation of flowering time in the autonomous flowering pathway by repressing FLOWERING LOCUS C expression.
18	Protein indeterminate-domain 2-like				12	0E+00	Plays a redundant role with IDD14 in directing leaf and floral organ morphogenesis (PubMed:24039602).<br>Acts cooperatively with IDD15 to control silique and branche orientation (PubMed:24039602).
19	Auxilin-like protein 1				4 t2	8E-52	Promotes uncoating of clathrin-coated vesicles. May interact directly with clathrin.
